# Crystal structure of 4-sulfamoylanilinium di­hydrogen phosphate

**DOI:** 10.1107/S1600536814017462

**Published:** 2014-08-13

**Authors:** C. Muthuselvi, N. Mala, N. Srinivasan, S. Pandiarajan, R. V. Krishnakumar

**Affiliations:** a Department of Physics, Devangar Arts College, Aruppukottai 626 101, Tamil Nadu, India; b Department of Physics, Thiagarajar College, Madurai 625 009, Tamil Nadu, India

**Keywords:** crystal structure, 4-sulfamoylanilinium, di­hydrogen phosphate, hydrogen bonding, sulfanilamide derivatives, sulfa drugs

## Abstract

In the crystal structure of the title mol­ecular salt, C_6_H_9_N_2_O_2_S^+^·H_2_PO_4_^−^, the sulfomylalinium cations and the di­hydrogen phosphate anions form independent [100] chains through N_s_—H⋯O (s = sulfamo­yl) and O—H⋯O hydrogen bonds, respectively. The chains are cross-linked by N_a_—H⋯O (a = amine) hydrogen bonds, generating (010) sheets. Two C—H⋯O hydrogen bonds involving diametrically opposite C atoms in the benzene ring of the cation as donors form chains parallel to [202] in which P=O and P—OH groups are acceptors. Together, these inter­actions lead to a three-dimensional network.

## Related literature   

For background to sulfa drugs, see: Topacli & Kesimli (2001 ▶); Gelbrich *et al.* (2007 ▶). For structures of other mol­ecular salts of the same cation, see: Anitha *et al.* (2013 ▶); Ravikumar *et al.* (2013 ▶); Pandiarajan *et al.* (2011 ▶); Zaouali Zgolli *et al.* (2010 ▶); Gelbrich *et al.* (2008 ▶); Chatterjee *et al.* (1981 ▶).
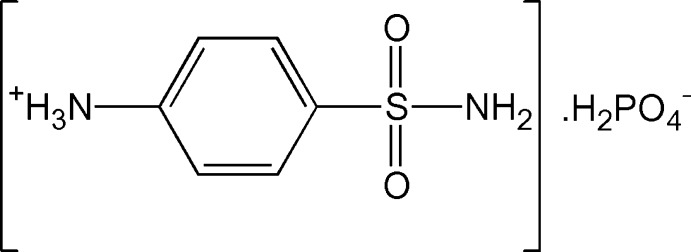


## Experimental   

### Crystal data  

C_6_H_9_N_2_O_2_S^+^·H_2_O_4_P^−^
*M* *_r_* = 270.20
Monoclinic, 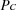

*a* = 4.8041 (7) Å
*b* = 10.8564 (15) Å
*c* = 10.3862 (15) Å
β = 101.067 (2)°
*V* = 531.62 (13) Å^3^
*Z* = 2
Mo *K*α radiation
μ = 0.47 mm^−1^
*T* = 294 K
0.28 × 0.18 × 0.10 mm

### Data collection  

Bruker SMART APEX CCD diffractometer
Absorption correction: multi-scan (*SADABS*; Bruker, 2001 ▶) *T*_min_ = 0.94, *T*_max_ = 0.99
6033 measured reflections
2512 independent reflections
2502 reflections with *I* > 2σ(*I*)
*R*_int_ = 0.018

### Refinement  

*R*[*F* ^2^ > 2σ(*F* ^2^)] = 0.021
*wR*(*F* ^2^) = 0.055
*S* = 1.06
2512 reflections
174 parameters
4 restraints
H atoms treated by a mixture of independent and constrained refinement
Δρ_max_ = 0.20 e Å^−3^
Δρ_min_ = −0.22 e Å^−3^
Absolute structure: Flack *x* determined using 1209 quotients [(*I* ^+^)−(*I* ^−^)]/[(*I* ^+^)+(*I* ^−^)] (Parsons & Flack, 2004 ▶)
Absolute structure parameter: 0.069 (16)

### 

Data collection: *SMART* (Bruker, 2001 ▶); cell refinement: *SAINT* (Bruker, 2001 ▶); data reduction: *SAINT*; program(s) used to solve structure: *SHELXS86* (Sheldrick, 2008 ▶); program(s) used to refine structure: *SHELXL2013* (Sheldrick, 2008 ▶); molecular graphics: *PLATON* (Spek, 2009 ▶); software used to prepare material for publication: *SHELXL2013*.

## Supplementary Material

Crystal structure: contains datablock(s) I. DOI: 10.1107/S1600536814017462/hb7251sup1.cif

Structure factors: contains datablock(s) I. DOI: 10.1107/S1600536814017462/hb7251Isup2.hkl

Supporting information file. DOI: 10.1107/S1600536814017462/hb7251Isup3.cml

**Fig. 1.**
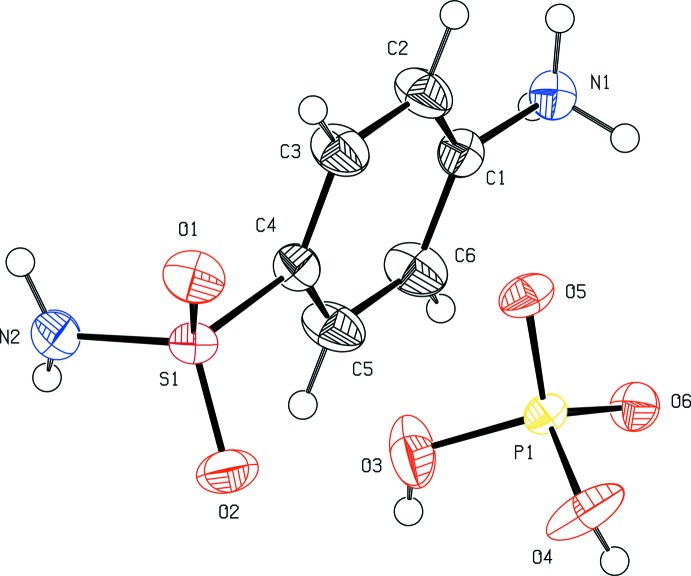
Molecular structure of (I) showing displacement ellipsoids drawn at the 50% probability level.

. DOI: 10.1107/S1600536814017462/hb7251fig1.tif
Mol­ecular structure of (I) showing displacement ellipsoids drawn at the 50% probability level.

**Fig. 2.**
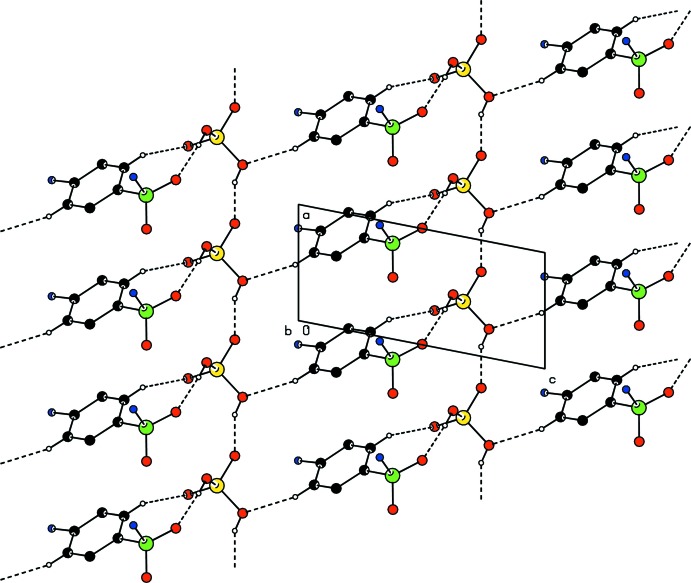
Hydrogen-bonding environment of the dihydrogen phosphate anion viewed along the b-axis. Other hydrogen bonds involving N atom and non-participating H atoms have been omitted for clarity.

. DOI: 10.1107/S1600536814017462/hb7251fig2.tif
Hydrogen-bonding environment of the di­hydrogen phosphate anion viewed along the b-axis. Other hydrogen bonds involving N atom and non-participating H atoms have been omitted for clarity.

CCDC reference: 1016950

Additional supporting information:  crystallographic information; 3D view; checkCIF report

## Figures and Tables

**Table 1:** Hydrogen-bond geometry (Å, °)

*D*—H⋯*A*	*D*—H	H⋯*A*	*D*⋯*A*	*D*—H⋯*A*
N1—H1*A*⋯O5^i^	0.87 (3)	1.89 (3)	2.760 (2)	174 (3)
N1—H1*B*⋯O6^ii^	0.90 (4)	1.96 (4)	2.856 (2)	170 (3)
N1—H1*C*⋯O6^iii^	0.86 (4)	2.00 (4)	2.855 (2)	175 (3)
N2—H2*A*⋯O3^iv^	0.86 (3)	2.25 (4)	3.076 (3)	161 (3)
N2—H2*B*⋯O2^v^	0.88 (4)	2.10 (4)	2.886 (3)	149 (3)
O3—H3*A*⋯O1^vi^	0.78 (2)	1.97 (2)	2.750 (3)	176 (4)
O4—H4*A*⋯O5^vi^	0.80 (3)	1.76 (3)	2.500 (2)	154 (5)
C3—H3⋯O6^v^	0.93	2.59	3.283 (3)	132
C6—H6⋯O4^i^	0.93	2.42	3.288 (3)	156

Symmetry codes: (i) 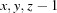; (ii) 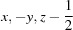; (iii) 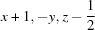; (iv) 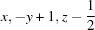; (v) 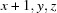; (vi) 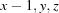.

## supplementary crystallographic information

### S1. Comment

Sulfanilamide (4-sulfamoyl aniline) and its derivatives known as sulfa drugs were used to treat bacterial infections (e.g. Gelbrich *et al.*, 2007). Sulfa drugs were successfully deployed as chemotherapeutic agents (Topacli & Kesimli, 2001).

The perchlorate (Anitha, *et al.*, 2013); nitrate, (Pandiarajan, *et al.*, 2011); sulfate (Ravikumar *et al.*, 2013) and hydrogen chloride (Zaouali Zgolli *et al.*, 2010) complexes of sulfanilamide were already been reported. The present report on phosphate salt of sulfanilamide is part of a series of x-ray investigations being carried out on sulfanilamide-inorganic acid complexes.

The crystal structure of the title compound contains a cation with a protonated amino group and the di­hydrogen phosphate anion C_6_H_9_N_2_O_2_S^+^.H_2_PO_4_^–^(Fig. 1). The crystal structure features a three-dimensional hydrogen bonding network formed through N—H···O, O—H···O and C—H···O hydrogen bonds. The sulfomylalinium cations and the phosphate anions form independent chains through N—H···O [ N1—H1A···O5 ; N1—H1B··· O6 ; N1—H1C···O6 ; N2—H2A···O3 and N2—H2B···O2]. O—H···O [viz., O3—H3A,,,O1 (-1+x, y, z) and O4—H4A···O5 (-1+x, y, z)] hydrogen bonds along the shortest a-axis. Two C—H···O hydrogen bonds viz., C3—H3···O6 (1+x, y, z); C6—H6···O4 (x, y, -1+z) involving diametricaly opposite aryl carbon atoms in the benzene ring of the 4-sulfomylanilinum cation act as donors to form chains parallel to (202) in which P = O and P—OH are acceptors . This one dimensional chain is extended into a two dimensional layer parallel to the ac-plane through the same P—OH linking the adjacent phosophate anion and another P=O.

The overall picture of the complex inter­molecular inter­actions may be visualized in terms of sinple graph-set motifs; viz., R_2_^2^(9) motif generated through O3—H3A···O1 (-1+x, y, z) and C3—H3···O6 (1+x, y, z) hydrogen bonds, two R_6_^6^(26) motifs generated involving O3—H3A···O1 (-1+x, y, z) and C6—H6···O4(x, y, -1+z); O4—H4A···O5(-1+x,y,z) hydrogen bond inter­actions (Fig. 2).

### S3. Synthesis and crystallization

The title compound was synthesised by heating a mixture of sulphanilamide (3.4 g) and phospho­ric acid (0.5 ml of 98% concentration) in 20 ml of water as the stoichiometric ratio of 2:1 (at 60°C) under reflux for 1 h. The solution upon allowing to evaporate under room temperature yielded colourless needles of the title salt.

### Crystal data

**Table:**

C_6_H_9_N_2_O_2_S^+^·H_2_O_4_P^−^	*Z* = 2
*M**_r_* = 270.20	*F*(000) = 280
Monoclinic, *P**c*	*D*_x_ = 1.688 Mg m^−^^3^
*a* = 4.8041 (7) Å	Mo *K*α radiation, λ = 0.71073 Å
*b* = 10.8564 (15) Å	µ = 0.47 mm^−^^1^
*c* = 10.3862 (15) Å	*T* = 294 K
β = 101.067 (2)°	Needle, colourless
*V* = 531.62 (13) Å^3^	0.28 × 0.18 × 0.10 mm

### Data collection

**Table:**

Bruker SMART APEX CCD diffractometer	2502 reflections with *I* > 2σ(*I*)
φ and ω scans	*R*_int_ = 0.018
Absorption correction: multi-scan (*SADABS*; Bruker, 2001)	θ_max_ = 28.3°, θ_min_ = 1.9°
*T*_min_ = 0.94, *T*_max_ = 0.99	*h* = −6→6
6033 measured reflections	*k* = −14→14
2512 independent reflections	*l* = −13→13

### Refinement

**Table:**

Refinement on *F*^2^	H atoms treated by a mixture of independent and constrained refinement
Least-squares matrix: full	*w* = 1/[σ^2^(*F*_o_^2^) + (0.0415*P*)^2^ + 0.0212*P*] where *P* = (*F*_o_^2^ + 2*F*_c_^2^)/3
*R*[*F*^2^ > 2σ(*F*^2^)] = 0.021	(Δ/σ)_max_ < 0.001
*wR*(*F*^2^) = 0.055	Δρ_max_ = 0.20 e Å^−^^3^
*S* = 1.06	Δρ_min_ = −0.22 e Å^−^^3^
2512 reflections	Extinction correction: *SHELXL2013* (Sheldrick, 2013), Fc^*^=kFc[1+0.001xFc^2^λ^3^/sin(2θ)]^-1/4^
174 parameters	Extinction coefficient: 0.099 (8)
4 restraints	Absolute structure: Flack *x* determined using 1209 quotients [(*I*^+^)-(*I*^-^)]/[(*I*^+^)+(*I*^-^)] (Parsons & Flack, 2004)
Hydrogen site location: mixed	Absolute structure parameter: 0.069 (16)

### Special details

**Table:**

Geometry. All e.s.d.'s (except the e.s.d. in the dihedral angle between two l.s. planes) are estimated using the full covariance matrix. The cell e.s.d.'s are taken into account individually in the estimation of e.s.d.'s in distances, angles and torsion angles; correlations between e.s.d.'s in cell parameters are only used when they are defined by crystal symmetry. An approximate (isotropic) treatment of cell e.s.d.'s is used for estimating e.s.d.'s involving l.s. planes.

### Fractional atomic coordinates and isotropic or equivalent isotropic displacement parameters (Å^2^)

**Table:**

	*x*	*y*	*z*	*U*_iso_*/*U*_eq_	
C1	0.8098 (4)	0.14923 (16)	0.09044 (19)	0.0265 (3)	
C2	1.0153 (5)	0.1497 (2)	0.2035 (2)	0.0365 (5)	
H2	1.1473	0.0862	0.2199	0.044*	
C3	1.0231 (5)	0.2455 (2)	0.2922 (2)	0.0364 (5)	
H3	1.1623	0.2475	0.3680	0.044*	
C4	0.8220 (4)	0.33816 (17)	0.26728 (19)	0.0271 (4)	
C5	0.6183 (5)	0.3379 (2)	0.1532 (2)	0.0381 (5)	
H5	0.4862	0.4013	0.1368	0.046*	
C6	0.6116 (5)	0.2433 (2)	0.0639 (2)	0.0379 (5)	
H6	0.4759	0.2426	−0.0132	0.046*	
N1	0.7938 (4)	0.04600 (14)	−0.00122 (17)	0.0271 (3)	
N2	0.9589 (4)	0.57959 (17)	0.3299 (2)	0.0352 (4)	
O6	0.3160 (3)	0.08705 (13)	0.55239 (13)	0.0288 (3)	
O5	0.7279 (3)	0.12348 (14)	0.74157 (13)	0.0298 (3)	
O3	0.4839 (5)	0.30112 (15)	0.62757 (19)	0.0504 (5)	
O4	0.2474 (3)	0.1676 (2)	0.77341 (17)	0.0506 (5)	
O1	1.0051 (4)	0.42220 (15)	0.50222 (15)	0.0364 (3)	
O2	0.5320 (3)	0.48636 (17)	0.38326 (17)	0.0400 (4)	
P1	0.44104 (7)	0.16369 (4)	0.66908 (4)	0.02196 (12)	
S1	0.82319 (8)	0.45858 (4)	0.38175 (5)	0.02634 (13)	
H1A	0.760 (7)	0.072 (3)	−0.082 (3)	0.039 (7)*	
H1B	0.639 (8)	0.001 (3)	0.006 (3)	0.046 (8)*	
H1C	0.946 (8)	0.003 (3)	0.018 (3)	0.046 (8)*	
H2A	0.855 (8)	0.611 (3)	0.261 (3)	0.050 (9)*	
H2B	1.143 (8)	0.578 (3)	0.333 (3)	0.042 (8)*	
H3A	0.343 (6)	0.334 (3)	0.594 (4)	0.052 (10)*	
H4A	0.083 (6)	0.164 (4)	0.742 (4)	0.080 (14)*	

### Atomic displacement parameters (Å^2^)

**Table:**

	*U*^11^	*U*^22^	*U*^33^	*U*^12^	*U*^13^	*U*^23^
C1	0.0272 (8)	0.0273 (8)	0.0245 (8)	−0.0007 (7)	0.0037 (7)	0.0024 (6)
C2	0.0368 (11)	0.0306 (9)	0.0372 (10)	0.0102 (8)	−0.0053 (9)	−0.0037 (8)
C3	0.0362 (11)	0.0335 (10)	0.0336 (10)	0.0084 (8)	−0.0084 (8)	−0.0030 (8)
C4	0.0288 (9)	0.0255 (8)	0.0270 (8)	0.0011 (6)	0.0052 (7)	0.0007 (6)
C5	0.0395 (11)	0.0363 (10)	0.0344 (10)	0.0145 (8)	−0.0036 (9)	0.0009 (8)
C6	0.0402 (11)	0.0400 (11)	0.0288 (9)	0.0105 (8)	−0.0053 (7)	−0.0011 (8)
N1	0.0287 (8)	0.0275 (7)	0.0243 (7)	−0.0007 (6)	0.0035 (6)	0.0016 (6)
N2	0.0310 (9)	0.0275 (8)	0.0462 (10)	0.0021 (7)	0.0050 (7)	0.0048 (7)
O6	0.0273 (6)	0.0314 (7)	0.0262 (6)	−0.0009 (5)	0.0014 (5)	−0.0035 (5)
O5	0.0189 (6)	0.0402 (8)	0.0290 (6)	0.0020 (5)	0.0017 (5)	0.0063 (6)
O3	0.0514 (10)	0.0261 (7)	0.0608 (11)	−0.0079 (7)	−0.0212 (8)	0.0091 (7)
O4	0.0195 (7)	0.0980 (15)	0.0357 (9)	−0.0113 (7)	0.0086 (6)	−0.0243 (8)
O1	0.0389 (8)	0.0414 (8)	0.0276 (6)	0.0079 (7)	0.0031 (6)	0.0013 (6)
O2	0.0250 (7)	0.0473 (8)	0.0492 (9)	0.0050 (6)	0.0107 (6)	−0.0059 (7)
P1	0.01703 (19)	0.0251 (2)	0.0230 (2)	−0.00087 (15)	0.00186 (14)	−0.00013 (15)
S1	0.0230 (2)	0.0275 (2)	0.0287 (2)	0.00361 (16)	0.00527 (14)	0.00025 (15)

### Geometric parameters (Å, º)

**Table:**

C1—C2	1.381 (3)	N1—H1B	0.90 (4)
C1—C6	1.387 (3)	N1—H1C	0.86 (4)
C1—N1	1.463 (2)	N2—S1	1.6048 (19)
C2—C3	1.386 (3)	N2—H2A	0.86 (3)
C2—H2	0.9300	N2—H2B	0.88 (4)
C3—C4	1.384 (3)	O6—P1	1.4975 (14)
C3—H3	0.9300	O5—P1	1.5027 (13)
C4—C5	1.384 (3)	O3—P1	1.5774 (17)
C4—S1	1.7665 (19)	O3—H3A	0.78 (2)
C5—C6	1.380 (3)	O4—P1	1.5583 (16)
C5—H5	0.9300	O4—H4A	0.80 (3)
C6—H6	0.9300	O1—S1	1.4371 (16)
N1—H1A	0.87 (3)	O2—S1	1.4339 (15)
			
C2—C1—C6	121.19 (18)	C1—N1—H1C	109 (2)
C2—C1—N1	119.70 (18)	H1A—N1—H1C	113 (3)
C6—C1—N1	119.08 (18)	H1B—N1—H1C	111 (3)
C1—C2—C3	119.45 (19)	S1—N2—H2A	113 (2)
C1—C2—H2	120.3	S1—N2—H2B	116 (2)
C3—C2—H2	120.3	H2A—N2—H2B	117 (3)
C4—C3—C2	119.49 (19)	P1—O3—H3A	114 (3)
C4—C3—H3	120.3	P1—O4—H4A	113 (3)
C2—C3—H3	120.3	O6—P1—O5	115.53 (9)
C3—C4—C5	120.79 (18)	O6—P1—O4	112.17 (9)
C3—C4—S1	120.00 (16)	O5—P1—O4	105.78 (9)
C5—C4—S1	119.21 (15)	O6—P1—O3	110.94 (9)
C6—C5—C4	119.90 (19)	O5—P1—O3	104.87 (10)
C6—C5—H5	120.0	O4—P1—O3	106.92 (13)
C4—C5—H5	120.0	O2—S1—O1	118.66 (10)
C5—C6—C1	119.15 (19)	O2—S1—N2	106.94 (11)
C5—C6—H6	120.4	O1—S1—N2	107.39 (11)
C1—C6—H6	120.4	O2—S1—C4	106.61 (10)
C1—N1—H1A	110.5 (19)	O1—S1—C4	107.79 (10)
C1—N1—H1B	108 (2)	N2—S1—C4	109.21 (10)
H1A—N1—H1B	105 (3)		
			
C6—C1—C2—C3	0.3 (4)	C2—C1—C6—C5	−0.9 (3)
N1—C1—C2—C3	−177.7 (2)	N1—C1—C6—C5	177.0 (2)
C1—C2—C3—C4	1.0 (4)	C3—C4—S1—O2	−142.11 (19)
C2—C3—C4—C5	−1.7 (4)	C5—C4—S1—O2	37.8 (2)
C2—C3—C4—S1	178.21 (19)	C3—C4—S1—O1	−13.7 (2)
C3—C4—C5—C6	1.1 (4)	C5—C4—S1—O1	166.24 (18)
S1—C4—C5—C6	−178.88 (19)	C3—C4—S1—N2	102.68 (19)
C4—C5—C6—C1	0.3 (4)	C5—C4—S1—N2	−77.4 (2)

### Hydrogen-bond geometry (Å, º)

**Table:**

*D*—H···*A*	*D*—H	H···*A*	*D*···*A*	*D*—H···*A*
N1—H1*A*···O5^i^	0.87 (3)	1.89 (3)	2.760 (2)	174 (3)
N1—H1*B*···O6^ii^	0.90 (4)	1.96 (4)	2.856 (2)	170 (3)
N1—H1*C*···O6^iii^	0.86 (4)	2.00 (4)	2.855 (2)	175 (3)
N2—H2*A*···O3^iv^	0.86 (3)	2.25 (4)	3.076 (3)	161 (3)
N2—H2*B*···O2^v^	0.88 (4)	2.10 (4)	2.886 (3)	149 (3)
O3—H3*A*···O1^vi^	0.78 (2)	1.97 (2)	2.750 (3)	176 (4)
O4—H4*A*···O5^vi^	0.80 (3)	1.76 (3)	2.500 (2)	154 (5)
C3—H3···O6^v^	0.93	2.59	3.283 (3)	132
C6—H6···O4^i^	0.93	2.42	3.288 (3)	156

Symmetry codes: (i) *x*, *y*, *z*−1; (ii) *x*, −*y*, *z*−1/2; (iii) *x*+1, −*y*, *z*−1/2; (iv) *x*, −*y*+1, *z*−1/2; (v) *x*+1, *y*, *z*; (vi) *x*−1, *y*, *z*.
