# Supplementary figures and images for: Crystal structure of 4-sulfamoylanilinium di­hydrogen phosphate
Source: Acta Crystallogr Sect E Struct Rep Online. 2014 Aug 13;70(Pt 9):o997–8. doi: 10.1107/S1600536814017462 (PMC4186100; doi:10.1107/S1600536814017462)

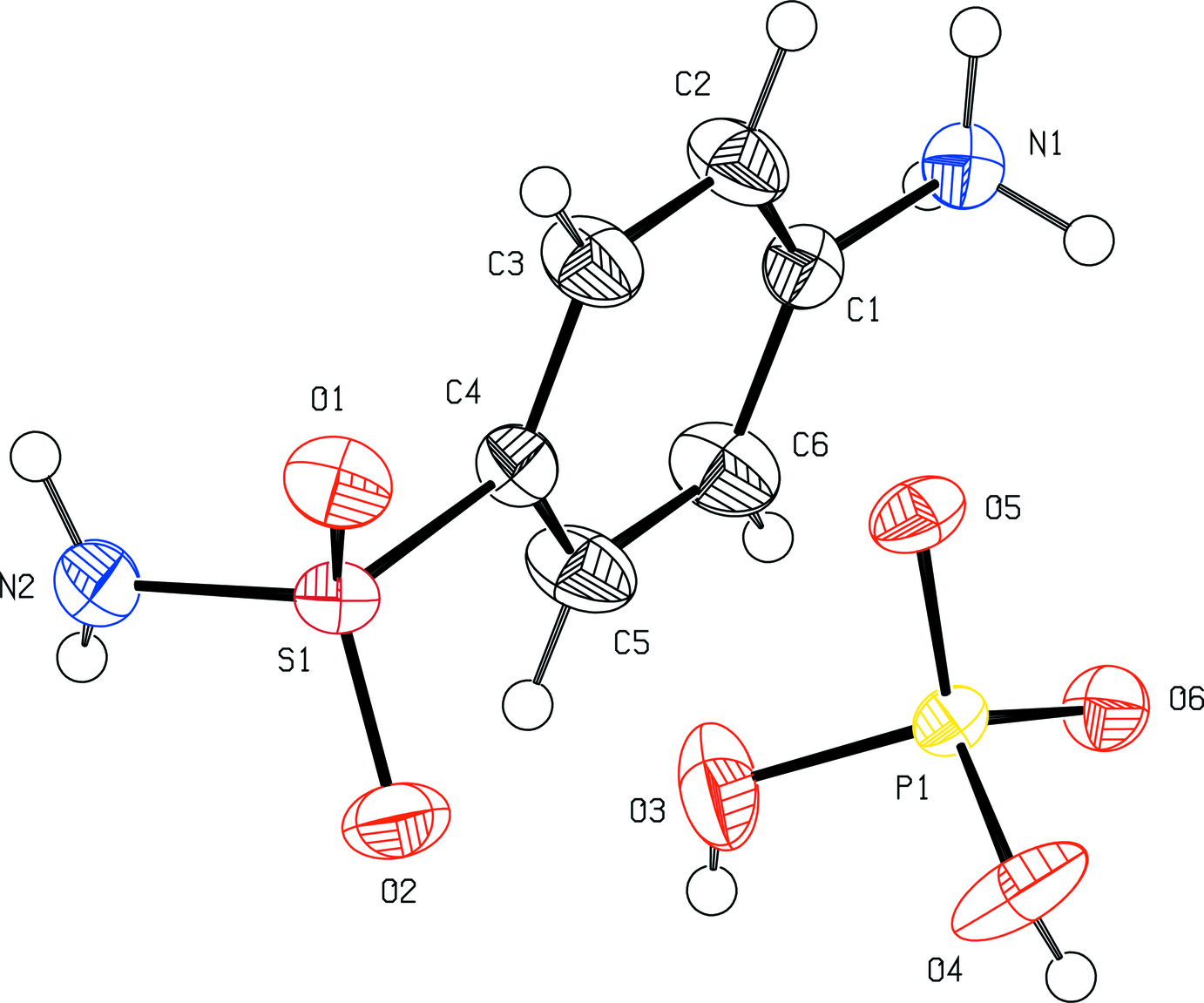

Supplement: Supplementary file 4 [file e-70-0o997-fig1.tif]

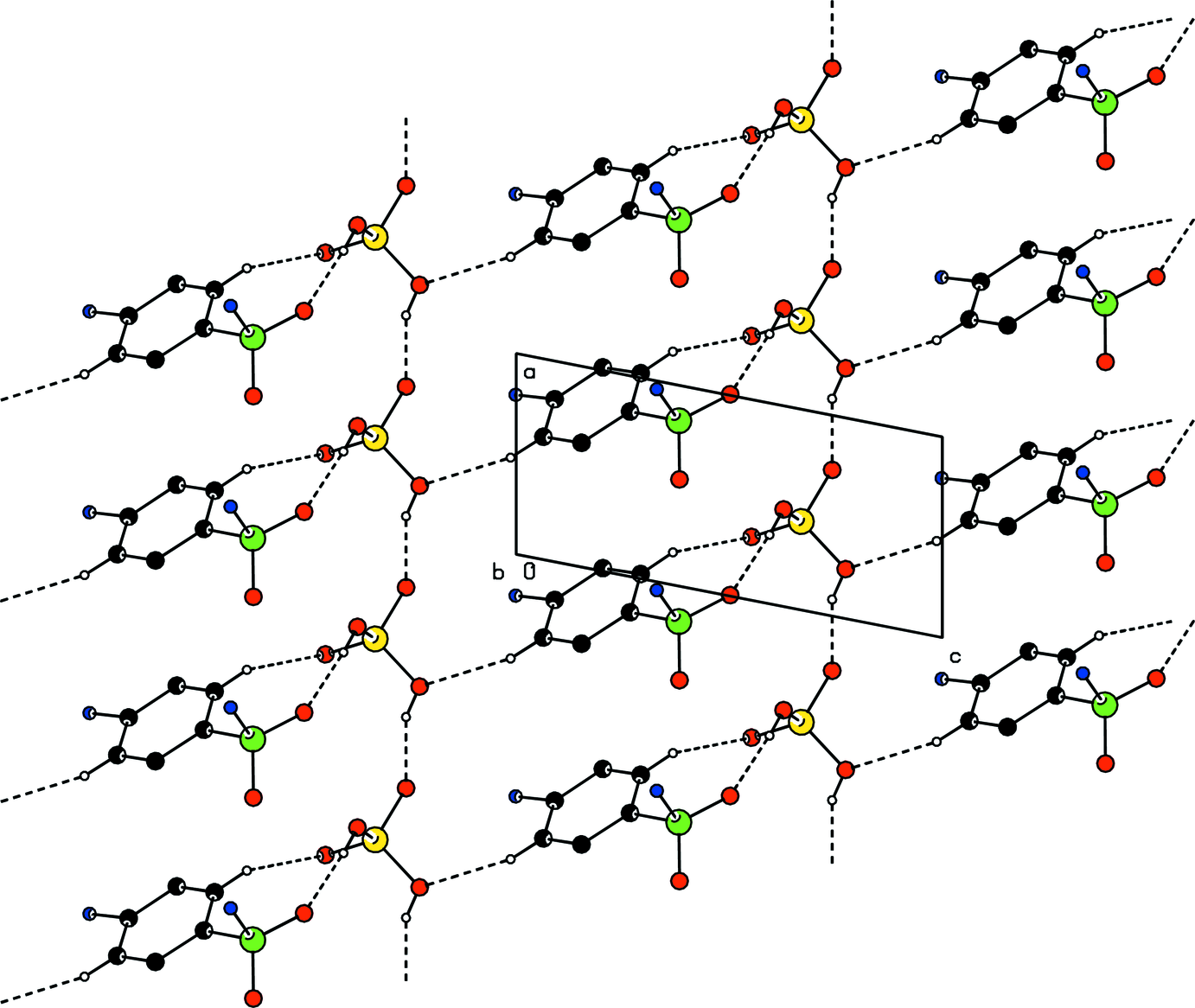

Supplement: Supplementary file 5 [file e-70-0o997-fig2.tif]
